# Clinical diagnostic value of contrast-enhanced ultrasound combined with microflow imaging in benign and malignant renal tumors: A retrospective cohort study

**DOI:** 10.17305/bb.2024.10236

**Published:** 2024-10-01

**Authors:** Xiufeng Kuang, Huiyang Wang, Weilu Chai, Huafang Yuan, Ting He, Mengya Shi, Tianan Jiang

**Affiliations:** 1 Department of Ultrasonography, Linping Campus, Second Affiliated Hospital, Zhejiang University School of Medicine, Hangzhou, Zhejiang, China; 2 Department of Ultrasound, The First Affiliated Hospital, College of Medicine, Zhejiang University, Hangzhou, Zhejiang, China; 3 Department of Ultrasonography, Affiliated Hangzhou First People’s Hospital, Zhejiang University School of Medicine, Hangzhou, Zhejiang, China

**Keywords:** Renal tumor, contrast-enhanced ultrasound (CEUS), microflow imaging (MFI), color Doppler flow imaging (CDFI), receiver operating characteristic (ROC) curve

## Abstract

This study aims to evaluate the clinical diagnostic value of contrast-enhanced ultrasound combined with microflow imaging (CEUS-MFI) in the differential diagnosis of benign and malignant renal tumors. All patients underwent contrast-enhanced ultrasound (CEUS), microflow imaging (MFI), color Doppler flow imaging (CDFI), and CEUS-MFI. The efficacies of these different diagnostic modalities in diagnosing benign and malignant renal tumors were evaluated by the Kappa consistency test and the receiver operating characteristic (ROC) curve, with pathological findings serving as the gold standard. CDFI, MFI, and CEUS-MFI all demonstrated higher blood flow in malignant tumors compared with benign tumors. Compared with benign tumors, CDFI detected a higher rate of punctate and linear Adler grade 2 and 3 blood flows in malignant tumors, as well as peripheral semicircular or annular blood flow. MFI identified a high rate of peripheral circumferential blood flow and irregular vascular morphology in malignant tumors, with most exhibiting Adler grade 3 blood flow. In addition, CEUS-MFI showed more dendritic or irregular Adler grade 2 or 3 blood flows in malignant renal tumors than MFI alone. Further analysis showed that CEUS-MFI had the highest consistency with pathological diagnosis (Kappa ═ 0.808). The ROC curve showed that the area under the curve (AUC) for CEUS-MFI in differentiating between benign and malignant lesions was 0.898, significantly outperforming other single diagnostic methods. With its capability to display microvascular information and assess overall pathological characteristics, MFI can accurately predict the nature of renal tumors and assist in surgical planning.

## Introduction

Renal neoplasms are common urological neoplasms. With the improvement of imaging technology, the detection rate of renal tumors has increased year by year [1]. These tumors can be benign or malignant [2]. Among the benign tumors, renal angiomyolipoma (RAML) is the most common, and can usually be monitored through periodic follow-up [2]. Renal cell carcinoma (RCC) is the most common primary renal malignancy, accounting for 90%–95% of malignant renal tumors, and often requires surgical resection [2, 3]. The discovery rate of asymptomatic RCC has increased in recent years [4]. However, the detection rate of RCC presenting with a “triad” of abdominal mass, low back pain, and hematuria is less than 15% of the discovery rate of renal cancer, and most are already in advanced stages at that time [4]. Due to the complexity of disease types, many subtypes, and overlapping imaging findings among subtypes of renal neoplasms, it is difficult to make a definitive diagnosis before surgery [5]. Precise preoperative prediction of the nature of renal tumors and their relationship with surrounding tissues can guide the treatment selection modalities and significantly improve the prognosis and quality of life of patients [6].

Commonly used renal tumor examination methods include computed tomography (CT), magnetic resonance imaging (MRI), and conventional ultrasound (CUS) [7]. CT and MRI have high diagnostic capabilities for renal tumors [7]. Of these, CT was strongly recommended as an imaging method for the diagnosis of renal tumors in the European Association of Urology Guidelines for RCC updated in 2019 [8]. However, CT and MRI are not suitable for all populations due to their high cost, radiation exposure, iodine or gadolinium allergies, etc. CUS has the advantages of safety, speed, lack of radiation exposure, reproducibility, and economic applicability, and is often used as the first-choice imaging method for renal tumor screening. CUS plays an important role in the early detection and diagnosis of renal tumors, though distinguishing between benign and malignant tumors can be challenging [9]. At present, it is a great challenge and of interest to identify effective and accurate imaging methods for predicting benign and malignant renal tumors before operation.

Contrast-enhanced ultrasound (CEUS) technology is known as “the third revolution of ultrasound” and has been widely used in the diagnostics of clinical renal masses [10]. It has been found that CEUS in renal malignancies mostly shows “fast progress,” “rapid regression,” and “hyperperfusion,” while benign tumors mostly exhibit “slow progression,” “slow regression,” and “hypoperfusion” [2]. However, the angiographic findings of some benign tumors overlap with RCC, making accurate diagnosis difficult [2]. Since neovascularization of tumors is essential for their occurrence and development [11], clearly displaying the blood flow in the mass is of great significance for the diagnosis of RCC [11]. Microflow imaging (MFI), a new vascular imaging technique, effectively separates low-velocity flow signals from tissue motion artifacts using intelligent algorithms, thereby improving the detection of low-speed blood flow signals and microvessels [12]. MFI has been reported to improve the diagnostic efficacy of renal solid masses in CEUS [13].

In this study, we compared the ultrasonographic features of CEUS in both benign and malignant renal tumors and the ability of MFI and color Doppler flow imaging (CDFI) to detect blood flow information of benign and malignant renal tumors. Additionally, we assessed the potential of contrast-enhanced ultrasound combined with microflow imaging (CEUS-MFI) in the diagnostics of renal tumors.

## Materials and methods

### Study subjects

The study retrospectively collected data from March 2021 to September 2022 on 97 patients (aged 28–75 years) with renal space-occupying lesions from The First Affiliated Hospital, College of Medicine, Zhejiang University. Of these, 55 had malignant lesions and 42 benign lesions.

Inclusion criteria were: 1) two-dimensional gray-scale ultrasound diagnosis of renal space-occupying lesions; 2) patients with single lesions; 3) usage of CEUS, CDFI, MFI, and CEUS-MFI; and 4) availability of surgical treatment and pathological results.

Exclusion criteria were: 1) patients who received intervention before the operation; 2) pregnant and lactating women; and 3) patients with contraindications for CEUS, such as severe cardiopulmonary dysfunction or allergy to contrast media.

### Instruments

CEUS, CDFI, MFI, and CEUS-MFI were performed by Philips Epiq7 ultrasound diagnostic instrument. The instrument was equipped with a 5-1 convex array probe with a frequency range of 1–5 MHz. MFI, CDFI, and CEUS-MFI imaging software were used. For CEUS, the mechanical index of 0.07 was set. SonoVue dry powder from Bracco (59 mg/syringe) was used as the ultrasound contrast agent, diluted to 5 mL with normal saline, and repeatedly oscillated to create a milky white microbubble suspension.

### Examination

Firstly, the patients underwent gray-scale ultrasonography. They were instructed to fully expose their abdomen in the supine and lateral positions on the examination bed. The kidneys and tumor were scanned horizontally and longitudinally, recording basic information such as the location, size, boundary, echo, internal echo uniformity, and presence or absence of small fluid areas of the renal tumor. CDFI and MFI were used to observe the blood flow inside and around the tumor in multiple sections, with patients instructed to hold their breath if necessary. The sampling frame should contain approximately 1 cm of the tumor and its periphery. Static and dynamic images should be stored simultaneously. Next, CEUS was initiated by selecting the section that clearly shows the tumor and some normal renal cortex. After a bolus injection of 1.4 mL of contrast medium into the median cubital vein, the tube was flushed with 5 mL of normal saline. Dynamic images of the entire angiography process should be stored for at least 180 s.

After a 6-min interval, the preparation for the CEUS-MFI examination was performed. First, the maximum section of the lesion was taken into the CEUS mode, and real-time double-contrast (contrast and gray-scale images) was turned on CEUS-MFI mode was started under the CPA (color powerangio) button for adjustment and confirmation, and then switched back to the double-contrast mode under CEUS. A 1.4 mL of contrast medium was injected again and flushed with 5 mL normal saline. Meanwhile, the CEUS-MFI mode under was quickly started and timed using the CPA key. The most abundant section was scanned, and the images were adjusted. The entire process dynamic chart was stored for at least 180 s.

### Image interpretation criteria

Stored static and dynamic ultrasound images were retrospectively analyzed by two experienced ultrasound physicians in a double-blind manner. When the results were inconsistent, senior physicians were consulted until a consensus was reached. The CUS analysis included location, size, border, morphology, echo category, internal echo, anechoic areas, etc. CEUS analysis included Enhancement mode (Fast/Same/Slow forward), Peak intensity (High/Middle/Low enhancement), Peritumoral hyperenhancement Ring (Yes/No), Enhanced uniformity (Uniform/Uneven), Regression mode (Fast/Same/Slow backward), Post-fading strength (High/Middle/Low) in the tumor, etc. The analysis of CDFI and MFI included blood flow display rate, Adler classification, vascular morphology, and peripheral blood flow. Blood flow was graded using Adler’s semi-quantitative method [14, 15]: with grade 0 lesions showing no blood flow; grade 1 showing 1–2 punctate or thin rod-like vessels; grade 2 lesions showing 3–4 punctate vessels or 1 important vessel with a length close to or exceeding the radius of the lesion; and grade 3 lesions showing more than 5 punctate vessels or 2 longer vessels.

**Figure 1.**
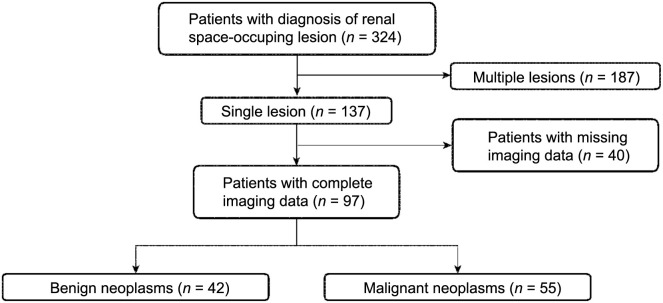
Flow diagram illustrating selection of study participants.

The vascular morphology included: 1) No blood flow: no blood flow signal in the lesion; 2) Spot-linear: stellar, short-line, or strip blood flow signals observed in the lesions; 3) Dendritic: the blood vessels in the lesions arranged in a dendritic distribution; and 4) Irregular shape: tortuous, ruptured, uneven thickness, and disordered arrangement of blood vessels in the lesions. Peripheral blood flow represented circular and semi-annular blood flow signals detected around the lesion were recorded as annular and semi-annular, respectively. The remainder was recorded as non-circular.

### Ethical statement

The research related to human use has been complied with all the relevant national regulations, institutional policies, and in accordance with the tenets of the Helsinki Declaration and has been approved by the Ethical Committee of The First Affiliated Hospital, College of Medicine, Zhejiang University with the approval number of IIT20210231B-R1 No. 026-Quick. All study subjects in the research were informed of the study content and signed informed consent forms.

### Statistical analysis

Data analysis was performed using SPSS 27.0. χ^2^ test was used to compare the enumerated data between groups. When theoretical frequency (T) was ≤ 5, the Fisher exact probability method was used. The consistency of diagnosis was evaluated by the Kappa test (consistency: Kappa value ≥ 0.75 was considered better, 0.75 > Kappa value ≥ 0.40 common, and Kappa value < 0.40 was considered poor). The receiver operating characteristic (ROC) curve was used to evaluate the diagnostic efficacy of different diagnostic methods in differentiating between benign and malignant renal lesions. The difference was statistically significant if *P* < 0.05.

## Results

### General patients’ data

A total of 97 lesions were included in this study, all of which were pathohistologically confirmed (Figure 1). Of these lesions, 42 were benign, including 37 angiomyolipomas, 3 eosinophilic tumors, and 2 epithelioid leiomyolipomas. There were 55 malignant lesions, including 48 clear cell carcinomas, 5 chromophobe carcinomas, and 2 papillary cell carcinomas. Of the 57 lesions with the largest diameter ≤ 4.0 cm, 29 were benign and 28 were malignant. Of the 40 lesions with the largest diameter > 4.0 cm, 13 were benign and 27 were malignant (Table 1).

**Table 1:** General data analysis of patients with renal tumors

**Parameters**		**Benign (*n* ═ 42)**	**Malignant (*n* ═ 55)**
Maximum diameter (cm)	≤ 4.0	29	28
	> 4.0	13	27
Tumor types	Renal angioleiomyolipoma	37	
	Renal oncocytoma	3	
	Renal epithelioid angiomyolipoma	2	
	Renal clear cell carcinoma		48
	Renal chromophobe cell carcinoma		5
	Renal papillary cell carcinoma		2

### CUS and CEUS characteristics of renal tumors

**Figure 2.**
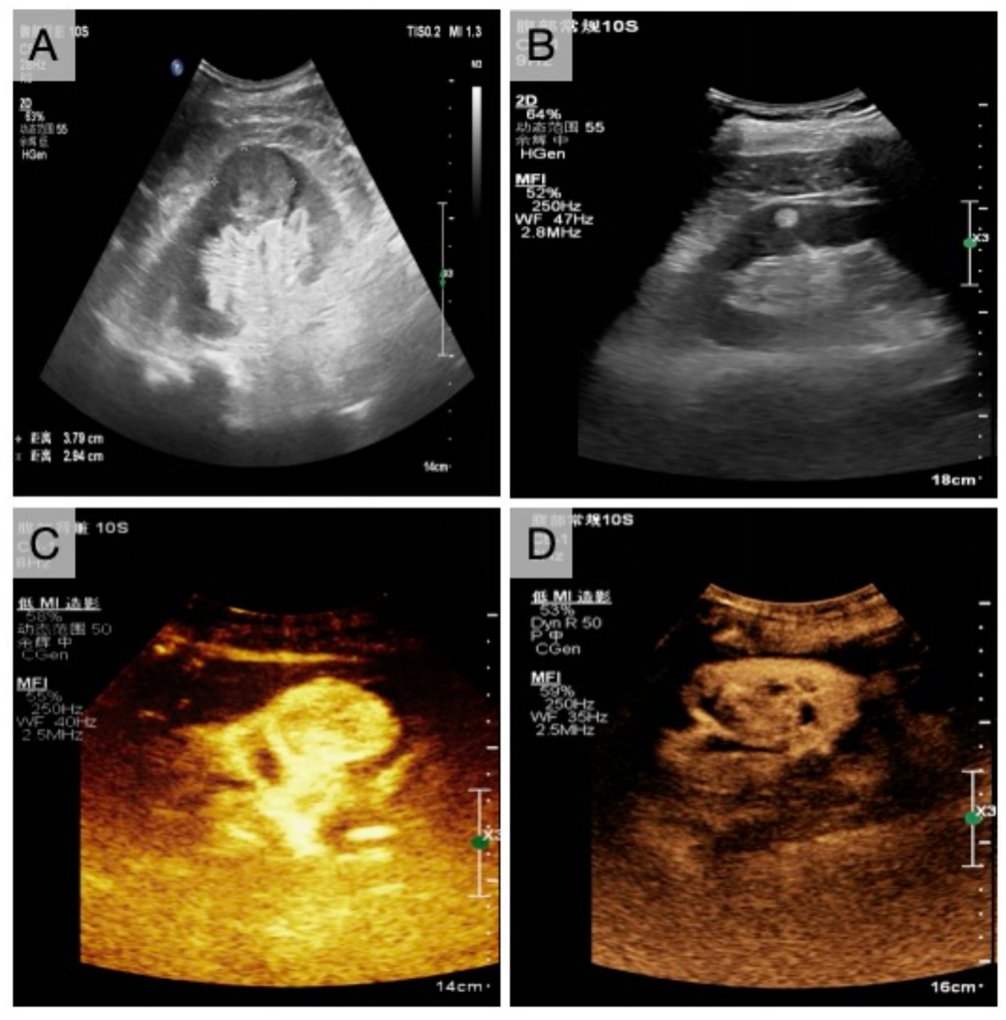
**Images of benign and malignant lesions in renal tumors by CUS and CEUS.** (A) Malignant and (B) benign tumors observed by CUS; (C) Malignant and (D) benign tumors observed by CEUS. CEUS: Contrast-enhanced ultrasound; CUS: Conventional ultrasound.

The characteristics of all benign and malignant lesions were observed by CUS, and the ultrasound images are shown in Figure 2A and 2B. Table 2 presents the corresponding statistical results. The distribution of 97 lesions showed no significant differences between benign and malignant tumors (*P* > 0.05). Most malignant tumors had clear boundaries, regular morphology, a low echo, uneven internal echo, and a high proportion of echo-free zones. The border of benign tumors was clear and regularly shaped, but the echo type was mainly hyperechoic, the internal echo homogeneous, and a low proportion of anechoic area. In addition, there were significant differences in the echo category, internal echo, morphology, and echo-free area between malignant and benign tumors (all *P* < 0.05).

**Table 2:** Comparison of CUS characteristics between benign and malignant tumors

**Parameters**		**Benign (*n* ═ 42)**	**Malignant (*n* ═ 55)**	***X* ^2^**	***P* value**
Position	Left kidney	21	28	0.008	0.929
	Right kidney	21	27		
Echo class	Hypoechoic	4	44	47.319	<0.001
	Hyperechoic	38	11		
Internal echo	Uniform	31	12	26.085	<0.001
	Nonuniform	11	43		
Border	Clear	36	46	0.079	0.779
	Unclear	6	9		
Form	Regular	41	45	5.914	0.021
	Irregular	1	10		
Anechoic area	Yes	11	49	39.934	<0.001
	No	31	6		

CUS: Conventional ultrasound.

Subsequently, the characteristics of all benign and malignant tumors by CEUS were further observed. The ultrasound images are shown in Figure 2C and 2D, and the corresponding statistical results are in Table 3. Most malignant tumors progress rapidly but fade slowly. These malignant tumors have uneven high enhancement at peak and are often accompanied by peritumoral high enhancement rings. Most tumors showed high enhancement after regression. Conversely, benign tumors usually presented with the same progression and regression, with uniform and equal enhancement at peak, but equal enhancement after regression, and few peritumoral hyperenhancement rings. In addition, there were significant differences in enhancement pattern, peak intensity, presence or absence of intratumoral hyperenhancement ring, regression pattern, and regression intensity between malignant and benign lesions (all *P* < 0.05). These results suggest that the CEUS features of the lesions can be used as an auxiliary diagnostic basis for benign and malignant renal tumors.

**Table 3:** Comparison of CEUS characteristics in benign and malignant tumors

**Parameters**		**Benign (*n* ═ 42)**	**Malignant (*n* ═ 55)**	***X* ^2^**	***P* value**
Enhancement mode	Fast forward	12	34	11.069	0.004
	Same forward	17	14		
	Slow forward	13	7		
Peak intensity	High enhancement	13	38	16.807	<0.001
	Middle enhancement	19	7		
	Low enhancement	10	10		
Peritumoral hyperenhancement	Yes	5	43	41.848	<0.001
	No	37	12		
Ring-enhanced uniformity	Uniform	28	30	1.455	0.228
	Uneven	14	25		
Regression mode	Fast backward	11	16	21.685	<0.001
	Same backward	26	11		
	Low backward	5	28		
Post-fading strength	High	10	28	7.395	0.025
	Middle	14	11		
	Low	18	16		

CEUS: Contrast-enhanced ultrasound.

### Blood flow characteristics of renal tumors using different diagnostic methods

Blood flow display in benign and malignant renal tumors was first detected by CDFI and MFI, respectively (Table 4). The flow visualization rates of CDFI, MFI, or CEUS-MFI in 97 renal tumors were statistically different between benign and malignant tumors (all *P* < 0.001). In addition, the flow visualization rates of CDFI, MFI, or CEUS-MFI in malignant tumors were 89.09%, 94.55%, or 96.36%, respectively, while those in benign tumors were only 47.62%, 45.24%, or 45.24%, respectively. Interestingly, CEUS-MFI showed a higher blood flow display rate in malignant tumors compared with CDFI and MFI.

**Table 4:** Comparison of blood flow display rate of renal tumors in different diagnostic methods

**Parameters**		**Benign (*n* ═ 42)**	**Malignant (*n* ═ 55)**	***X* ^2^**	***P* value**
CDFI	Blood flow display	20	49	19.947	<0.001
	No blood flow display	22	6		
	Display rate	47.62%	89.09%		
MFI	Blood flow display	19	52	29.510	<0.001
	No blood flow display	23	3		
	Display rate	45.24%	94.55%		
CEUS-MFI	Blood flow display	19	53	32.538	<0.001
	No blood flow display	23	2		
	Display rate	45.23%	96.36%		

CDFI: Color Doppler flow imaging; CEUS-MFI: Contrast-enhanced ultrasound combined with microflow imaging; MFI: Microflow imaging.

Subsequently, CDFI or MFI methods were used to evaluate blood flow characteristics in both benign and malignant renal tumors. As shown in Table 5, malignant tumors showed mostly punctate and linear grade 2 and grade 3 blood flow on CDFI, whereas the benign tumors displayed predominantly punctate and linear grade 1 or 2 blood flow or no blood flow. The detection rate of peripheral hemicyclic or annular blood flow in the malignant tumors was significantly higher than that in the benign tumors (*P* < 0.05). In addition, on MFI, renal tumors presenting with grade 3 blood flow were significantly more common among malignant than benign tumors (*P* < 0.001). In addition, the vascular morphology of malignant renal tumors was irregular, and the detection rate of peripheral circumferential blood flow was high. In contrast, the benign tumors were characterized by punctate linear flow and had a low detection rate of peripheral annular blood flow (*P* < 0.05, Table 5). Interestingly, CEUS-MFI showed similar lesion flow characteristics to MFI but showed more dendritic or irregular Adler grade 2 or 3 blood flow in malignant lesions. These results collectively suggested that CEUS-MFI more effectively displays the blood flow characteristics of renal malignant lesions than CDFI and MFI.

**Table 5:** Comparison of blood flow characteristics in benign and malignant renal tumors by CDFI, MFI, or CEUS-MFI

	**Parameters**		**Benign (*n* ═ 42)**	**Malignant (*n* ═ 55)**	***P* value**
CDFI	Adler grading	0	22	6	<0.001
		1	7	13	
		2	8	24	
		3	5	12	
	Blood vessel morphology	N/D	22	6	<0.001
		Dotted line	19	34	
		Arborization	0	9	
		Irregularity	1	6	
	Peripheral blood flow	N/D	31	30	0.038
		Semi-ring	5	19	
		Ring	6	6	
MFI	Adler grading	0	23	3	<0.001
		1	6	11	
		2	7	10	
		3	6	31	
	Blood vessel morphology	N/D	23	3	<0.001
		Dotted line	16	18	
		Arborization	0	12	
		Irregularity	3	22	
	Peripheral blood flow	N/D	28	6	<0.001
		Semi-ring	2	5	
		Ring	12	44	
CEUS-MFI	Adler grading	0	23	2	<0.001
		1	4	9	
		2	8	10	
		3	7	34	
	Blood vessel morphology	N/D	23	2	<0.001
		Dotted line	15	13	
		Arborization	0	16	
		Irregularity	4	24	
	Peripheral blood flow	N/D	26	4	<0.001
		Semi-ring	8	9	
		Ring	8	42	

CDFI: Color Doppler flow imaging; CEUS-MFI: Contrast-enhanced ultrasound combined with microflow imaging; MFI: Microflow imaging.

### Consistency analysis and diagnostic efficiency of different diagnostic methods in differentiating between benign and malignant renal tumors

Based on the different ultrasound and blood flow characteristics of benign and malignant renal tumors, we further analyzed the consistency of different diagnostic modalities and pathological findings in distinguishing between the two. As shown in Table 6, the kappa coefficients for CEUS, MFI, and CDFI were 0.602, 0.638, and 0.644, respectively (all *P* < 0.001). In addition, the kappa coefficient of CEUS-MFI (0.808) was significantly higher than either of the individual diagnostic modalities. These values suggest a higher diagnostic consistency of CEUS-MFI compared with CEUS, MFI, or CDFI alone in diagnosing benign and malignant renal tumors.

**Table 6:** Consistency analysis between different examination methods and pathological diagnosis results

**Parameters**		**Pathological diagnosis**		**Kappa**	***P* value**
		+	−		
RCC angiography	+	45	9	0.602	<0.001
	−	10	33		
MFI	+	49	11	0.638	<0.001
	−	6	31		
CDFI	+	46	8	0.644	<0.001
	−	9	34		
CEUS-MFI	+	53	7	0.808	<0.001
	−	2	35		

CDFI: Color Doppler flow imaging; CEUS-MFI: Contrast-enhanced ultrasound combined with microflow imaging; MFI: Microflow imaging; RCC: Renal cell carcinoma.

**Figure 3.**
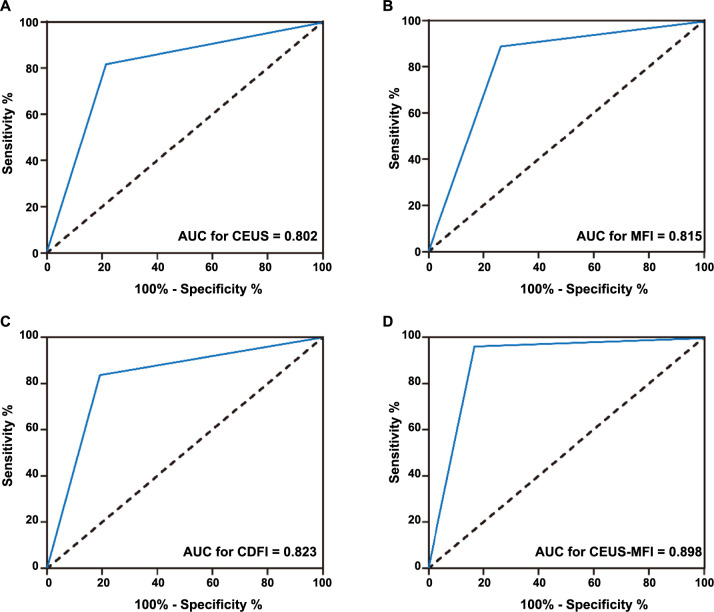
**ROC curve of benign and malignant renal tumors by different diagnostic methods.** ROC: Receiver operating characteristic; AUC: Area under the curve; CEUS: Contrast-enhanced ultrasound; MFI: Microflow imaging; CDFI: Color Doppler flow imaging; CEUS-MFI: Contrast-enhanced ultrasound combined with microflow imaging.

The diagnostic efficacy of different diagnostic modalities for benign and malignant renal tumors is shown in Figure 3 and Table 7. The area under the curve (AUC) of CEUS, MFI, and CDFI was 0.802, 0.815, and 0.823, the sensitivity was 81.8%, 89.1%, and 83.6%, and the specificity was 78.6%, 73.8%, and 90.0%, respectively. Interestingly, CEUS-MFI had the highest diagnostic AUC (0.898), with sensitivity and specificity of 96.4% and 83.3%. In addition, the diagnostic accuracy and F1-score of CEUS-MFI were significantly higher than that of CEUS, MFI, and CDFI (accuracy: 90.7% vs 80.4%, 82.5%, 82.5%; F1-score: 92.17% vs 82.54%, 85.24%, 84.39%, all *P* < 0.001). These results suggest that CEUS-MFI has the highest diagnostic efficacy in distinguishing between benign and malignant renal tumors.

**Table 7:** Differential diagnosis of benign and malignant renal tumors by different examination methods

**Parameters**	**CEUS**	**MFI**	**CDFI**	**CEUS-MFI**
AUC (95% CI)	0.802 (0.709, 0.895)	0.815 (0.722, 0.907)	0.823 (0.734, 0.912)	0.898 (0.825, 0.971)
Sensitivity	81.80%	89.10%	83.60%	96.40%
Specificity	78.60%	73.80%	90.00%	83.30%
True positive	83.30%	81.70%	85.20%	88.30%
False positive	16.70%	18.30%	14.80%	11.70%
True negative	76.70%	83.80%	79.10%	94.60%
False negative	23.30%	16.20%	20.90%	5.40%
Accuracy	80.40%	82.50%	82.50%	90.70%
F1-score	82.54%	85.24%	84.39%	92.17%

CDFI: Color Doppler flow imaging; AUC: Area under the curve; CEUS: Contrast-enhanced ultrasound; CEUS-MFI: Contrast-enhanced ultrasound combined with microflow imaging; MFI: Microflow imaging.

## Discussion

As a low-cost and well-tolerated imaging method, CEUS can dynamically display microcirculatory perfusion and tumor vasculature in real time, and its value in differentiating between benign and malignant renal tumors has been widely recognized [16, 17]. However, the specificity of CEUS in diagnosing benign and malignant renal tumors is relatively low due to the overlapping ultrasound findings across different pathological types, which cannot be judged solely from the contrast-enhanced mode [17]. In the CEUS mode, the hyperenhancement ring around the lesion can reflect the capillary condition in the pseudocapsule around the renal tumor with high specificity [8, 18]. In practice, however, the detection rate of ring enhancement is low, leading to the relatively low sensitivity of CEUS for differential diagnosis [19]. CEUS-MFI is the latest microvascular imaging technique. It fuses MFI on top of CEUS without breaking the microbubble mode of contrast medium, preserving the advantages of CEUS and MFI, while having a high spatial resolution, very low motion artifacts, and high-frequency imaging [20]. In addition, CEUS-MFI can dynamically display the morphological and architectural characteristics of fine blood flow in lesions in real time, especially with a high detection rate for annular blood flow in tumors [20]. There are few reports on CEUS-MFI in the diagnosis of renal tumors. Therefore, this study compared single CEUS, MFI, and CDFI, and applied the CEUS-MFI technique to renal tumors, and explored its value in differentiating between benign and malignant renal tumors.

The epidemiology of benign and malignant renal tumors varies [21]. RAML is the most common benign renal tumor, occurring mostly in patients with tuberous sclerosis, and most lesions are <4 cm in size [22]. Clear cell carcinoma is the most common malignancy from renal parenchyma, followed by papillary RCC and chromophobe cell carcinoma [21]. The results of this study showed that the largest diameter of benign renal tumors was less than 4 cm, with RAML being the most prevalent type. Clear cell carcinoma accounts for the majority of malignant renal tumors, consistent with the epidemiology of renal tumors [4]. CEUS can observe the enhancement mode, regression mode, degree of enhancement, regression intensity, enhancement uniformity, and whether there is a high enhancement ring in the tumor [4]. The results of this study showed that the largest diameter of the malignant tumors was higher than that of the benign tumors. This may be due to the growth rate and echo of the tumor. Malignant tumors have a fast growth rate, and are asymptomatic isoechoic or hypoechoic in the early stage, making them difficult to detect when smaller in size. In some cases, patients with clinical symptoms have a larger tumor volume when seeking medical treatment. In contrast, benign tumors grow slowly and are mostly hyperechoic due to their fat content. They are typically incidentally detected when they are smaller in size, so the maximum diameter of detected benign renal tumors is relatively small.

Subsequently, we compared and analyzed the differences in CUS and CEUS characteristics between benign and malignant tumors. Benign and malignant renal tumors in this study mostly presented as well-circumscribed round-like lesions, consistent with previous findings [17]. This may be related to the growth pattern of renal tumors. Benign renal tumors grow slowly and are non-invasive into the surrounding normal renal tissues, thus clearly demarcated from the surrounding tissues. Conversely, malignant renal tumors have a fast growth rate and limited space, restricting their growth. When the size of a kidney tumor increases to a certain extent, it can compress the surrounding kidney tissue. This compression may lead to ischemic necrosis, followed by the formation of fibrous tissue [23]. These fibrous tissues have been shown to be characteristic of RCC [24]. In addition, the echogenic category of the tumor, whether the internal echo is homogeneous, and the presence or absence of liquid anechoic areas are related to the histological subtypes of benign and malignant renal tumors [25]. The results of this study showed that there were significant differences between the two groups in terms of echo category, internal echo, and presence or absence of liquid anechoic areas. Benign renal tumors often showed homogeneous hypoechoic areas, while malignant renal tumors showed heterogeneous hypoechoic areas with fluid anechoic area. Additionally, some benign renal tumors > 4 cm showed uneven hyperechoic areas with fluid anechoic areas. In addition, the benign tumors showed synchronous and homogeneous enhancement, with low enhancement after regression. Malignant tumors showed fast progression and slow regression, uneven high enhancement, and high enhancement after regression. This variation is linked to the extensive neovascularization—both in number and diameter—observed in certain malignant tumors like clear cell carcinoma, which also benefit from a rich blood supply following the formation of arteriovenous fistulas in normal vessels. Conversely, other malignancies such as papillary renal cell carcinoma are characterized by smaller vessel diameters, lower blood flow density, and slower velocity. These results suggest that the differential CEUS features of renal tumors can be used as one of the diagnostic modalities for the differential diagnosis of benign and malignant tumors.

The occurrence and development of renal tumors are inseparable from the neovascularization within the tumors [26]. Microvessel density has been shown to be associated with tumor grade, metastasis, and prognosis [26]. Relevant pathological studies have also shown that the number and grade of blood flow are significantly higher in malignant renal tumors than in benign renal tumors [27, 28]. Therefore, investigating a method to identify the microvascular system of renal tumors is vital for early diagnosis and differentiation between benign and malignant tumors.

In this study, CDFI and MFI were used to detect blood flow display and blood flow characteristics in benign and malignant renal tumors. Both CDFI and MFI showed higher blood flow in malignant than in benign tumors. In malignant tumors, CDFI or MFI blood flow was mostly Adler grade 2 or 3, whereas benign tumors predominantly showed Adler grade 0 or 1, consistent with previous findings [28]. This is possibly due to the size and neovascularization of the tumor. Compared with normal vessels, neovascularization in renal malignancies is characterized by increased number, increased diameter, compression, displacement, structural disturbance, and distorted course, as well as irregular secondary vascular branches and even venous fistulas [29]. While benign renal tumors also have neovascularization, their number, diameter, and vascular branches are relatively small [30]. In addition, the vascular morphology of malignant tumors detected by CDFI was mainly punctate and linear, and the peripheral blood flow was mostly circular or semi-circular, which is consistent with previous studies [30]. Interestingly, CDFI predominantly detected grade 2 blood flow in malignant tumors, whereas MFI showed more grade 3 blood flow, due to MFI’s ability to detect smaller, low-velocity blood flow signals. This indicates that in the small malignant lesions, the blood vessels are thin and the blood flow velocity is slow. MFI can recognize the low-velocity blood flow signal and the delicate branches of blood vessels which cannot be recognized by CDFI. Moreover, CEUS combined with MFI can display the blood flow signals of renal malignant lesions to a greater extent than MFI alone, indicating that CEUS-MFI has a greater advantage in the analysis of blood flow characteristics of benign and malignant renal tumors.

Finally, we analyzed the diagnostic efficacy of CEUS, MFI, CDFI, and CEUS-MFI in benign and malignant renal tumors and their concordance with pathological findings. The ROC AUC of CEUS, MFI, CDFI, and CEUS-MFI was 0.802, 0.815, 0.823, and 0.898, respectively, and the diagnostic accuracy was 80.4%, 82.5%, 82.5%, and 90.7%, respectively. The concordance between pathological diagnosis and four different diagnostic methods of CEUS, MFI, CDFI, and CEUS-MFI was 0.602, 0.638, 0.644, and 0.808, respectively, indicating that CEUS-MFI is more effective than a single diagnostic method. These results suggest that CEUS-MFI has the highest diagnostic efficacy in differentiating between benign and malignant renal tumors. Interestingly, the diagnostic specificity of MFI was only 73.8%, as it could detect finer and low-velocity blood flow signals, and lead to misdiagnosis in some benign lesions with abundant blood flow. CEUS improved the sensitivity, specificity, and accuracy of MFI detection. This is because lesions that usually present as well-circumscribed, well-defined homogeneous hyperechoic morphology on CEUS tend to be benign, whereas lesions presenting as heterogeneous hypoechoic areas with anechoic interior often tend to be malignant. However, findings on CEUS of some atypical benign and malignant renal tumors overlap and it is difficult to differentiate between them. MFI can clearly display the small blood flow signals within and around the tumor and can reflect the blood supply, neovascularization, and the blood flow of the tumor and its surrounding tissues in real time, aiding in the determination of the tumor’s nature.

This study has the following limitations: 1) This study is a single-center study with limited sample size and possible biased results; 2) Space constraints prevented statistical analysis of CEUS characteristics, as well as CDFI and MFI blood flow characteristics, for renal tumors of varying sizes; and 3) CEUS features, CDFI, and MFI blood flow characteristics among different tissue subtypes of renal tumors were not studied.

## Conclusion

In summary, CEUS combined with MFI can thoroughly assess the characteristics of renal tumors based on the differences of CEUS characteristics and the MFI’s depiction of tumor microvascular information. This provides a basis for distinguishing between benign and malignant renal tumors more accurately, enhancing sensitivity and specificity. Moreover, it aids in objectively determining the nature of renal tumors in a clinical environment.

## Data Availability

Data sharing is not applicable to this article as no datasets were generated or analyzed during the current study.
